# Gyroscope-Based Video Stabilization for Electro-Optical Long-Range Surveillance Systems

**DOI:** 10.3390/s21186219

**Published:** 2021-09-16

**Authors:** Petar D. Milanović, Ilija V. Popadić, Branko D. Kovačević

**Affiliations:** 1 School of Electrical Engineering, University of Belgrade, Bul. Kralja Aleksandara 73, 11120 Belgrade, Serbia; kovacevic_b@etf.bg.ac.rs; 2 Vlatacom Institute of High Technologies, Milutina Milankovica 5, 11070 Belgrade, Serbia; ilija.popadic@vlatacom.com

**Keywords:** video stabilization, MEMS gyroscope, electro-optical long-range surveillance systems

## Abstract

Video stabilization is essential for long-range electro-optical systems, especially in situations when the field of view is narrow, since the system shake may produce highly deteriorating effects. It is important that the stabilization works for different camera types, i.e., different parts of the electromagnetic spectrum independently of the weather conditions and any form of image distortion. In this paper, we propose a method for real-time video stabilization that uses only gyroscope measurements, analyze its performance, and implement and validate it on a real-world professional electro-optical system developed at Vlatacom Institute. Camera movements are modeled with 3D rotations obtained by integration of MEMS gyroscope measurements. The 3D orientation estimation quality depends on the gyroscope characteristics; we provide a detailed discussion on the criteria for gyroscope selection in terms of the sensitivity, measurement noise, and drift stability. Furthermore, we propose a method for improving the unwanted motion estimation quality using interpolation in the quaternion domain. We also propose practical solutions for eliminating disturbances originating from gyro bias instability and noise. In order to evaluate the quality of our solution, we compared the performance of our implementation with two feature-based digital stabilization methods. The general advantage of the proposed methods is its drastically lower computational complexity; hence, it can be implemented for a low price independent of the used electro-optical sensor system.

## 1. Introduction

The use of video acquisition devices has increased dramatically in the last ten years. Video cameras have become popular in the consumer sphere as well as in industrial and military applications. Unwanted parasitic vibration in video sequences exists in all video systems and significantly degrades their value and performance [1].

Digital video stabilization is the process of removing unwanted movements from video sequences and creating outputs that ideally contain only smooth desired movements. It should provide a technique for image analysis and processing that will remove image shaking, resulting in a stable video output, which is easier to be followed by user. Conceptually, video stabilization is accomplished by forming a rectangular clip of an original video sequence whose position within the frame varies by tracking unwanted camera movements. Ideally, the clip moves along with the recorded content such that the content inside the clip remains stable. Estimation of frame-to-frame clip position (or corresponding camera movement) can be performed in three ways:

1. By using direct processing of the recorded video (typically by tracking the corresponding features in the recorded video, from frame to frame, e.g., [2,3,4,5,6,7]);
2. By using direct 3D measurements of camera movement using a gyroscope or an inertial measurement unit (IMU) device [8,9,10];
3. By combining/fusion of these two approaches [11,12,13,14,15].

**Figure 1:**
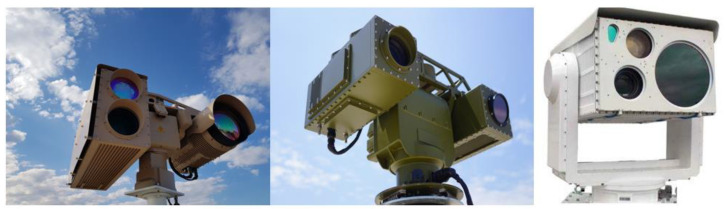
Vlatacom electro-optical long-range surveillance system.

In this paper, we present a video stabilization method for Vlatacom electro-optical long-range surveillance systems, shown in Figure 1. These systems usually consist of two or three different electro-optical systems (cameras) placed on a pan/tilt positioner. Each camera (image sensor) covers a different part of the electromagnetic spectrum, i.e., visible light, or infrared—(SWIR, MWIR or LWIR). Pan/tilt positioner is a servo-driven two-axis device that is designed for long-distance video surveillance systems that require azimuth and elevation rotation with high accuracy and angular velocity. Complete video signal processing, including stabilization, is performed only on the video signal processing module (vVSP) [16]. It is integrated into the housing together with the corresponding camera.

Stabilization is essential for most long-range multi-sensor camera systems with a pan/tilt because the effect of system shake is more pronounced when using larger focal lengths or narrower fields of view (FOV). The pan/tilt usually possesses a system for mechanical gyroscope-based stabilization, but due to the imperfection of the gyroscope pan/tilt drifts, such observed objects move from the center of the screen with a tendency to disappear. As the viewing angle decreases, this effect becomes more noticeable. The pan/tilt drift is described in more detail in [17]. Furthermore, these electro-optical systems typically have an image feature-based algorithm for direct digital video stabilization. However, the performance of these algorithms is poor when observing scenes without clearly expressed shapes (edges), i.e., the sea, desert, sky, scenes with moving objects, under bad weather conditions (rain, snow, fog, haze) or if any form of image distortion is presented. This is because the features extraction and frame-to-frame tracking of the corresponding features are difficult or impossible in these scenarios. The goal of our stabilization is to overcome the aforementioned issues.

Since stabilization needs to be independent of observing scenes, weather conditions and image distortions, in our work, we focus on a method based on gyroscope measurements. We model the camera movement with 3D rotation relative to the global coordinate system. Three-dimensional orientation is obtained by integration of gyroscope samples. The presence of possible unwanted camera translations is less common in practice. When it exists, the effect of small translations on the recorded video is typically negligible compared to the effect of small rotations. In addition, a possible estimation of 2D scene-to-frame motion using a 3D accelerometer is technically more demanding (it requires depth estimation) and prone to larger errors due to double integration [8].

Gyroscope video stabilization is typically accomplished in three stages [10]:

1. Frame to frame estimation of camera orientation change based on gyroscope measurements;
2. Filtering of the estimated camera orientation to obtain unwanted camera motion;
3. Transformation (homography) of the recorded frames based on the estimated unwanted motion and calibrated 3D to 2D transformation.

**Figure 2:**
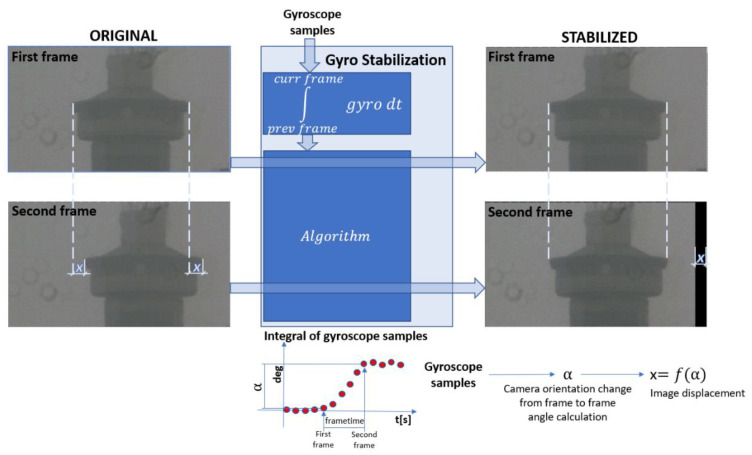
Illustrative representation of video stabilization using gyroscope measurements.

Figure 2 illustrates gyroscope video stabilization. Gyroscope measures angular velocity of the camera. Measurements are integrated to obtain angular orientation in 3D space. A stabilization algorithm takes these values, transforms them to the image plane, estimates movements in the image space and performs image warping to obtain a stabilized image.

The state-of-the-art algorithms based on hardware solutions, mainly gyroscope based stabilization, are developed for wide-angle cameras such as cameras in smartphones [8,9,10]. The movement of smartphones or similar devices are large compared to the movements of electro-optical systems; thus the gyroscope cannot be the same. Besides movements of camera that needs to be stabilized, there are also wanted movements, e.g., the movement of the person carrying the camera that authors want to keep. Steven Bell et al. [8] designed a non-linear filter to separate wanted and unwanted movements. They also, similar to Alexander Karpenko et al. [10], developed solution for rolling shutter correction. Hannes Ovren et al. [18] propose auto-calibration technique to determine the time offset and relative pose between gyroscope and camera. Chao Jia et al. [9] proposed IIR-based and UKF-based (unscented Kalman filter) methods to smooth camera motion sequence. They also dealt with black border problem.

In this paper, we propose a suitable real-time algorithm, analyze its performance, implement and validate it on the multi-spectral camera system for surveillance. Since the estimation of 3D camera system orientation is performed by directly integrating gyro measurements, we provide an analysis of the orientation estimation quality depending on the gyro sensitivity, measurement noise and drift stability. We then propose a method for estimating the unwanted motion using interpolation in the quaternion domain, which is subsequently transformed in the 2D image coordinate system. We also propose methods for eliminating disturbances originating from gyro bias instability and noise. The parameters used for this transformation are selected and calibrated using a non-convex optimization technique (which can be implemented offline). When calibrated, our algorithm has low computational complexity and can be implemented on hardware components (such as FPGA, CPU), independently of the used camera system. We finally verify and discuss the clear advantages of our approach on several characteristic real-life examples, and we prepare a performance comparison with Google and RFEL feature-based digital stabilization.

The main contribution of this paper can be summarized into the following:

- A general stabilization algorithm has been adopted in the long-range electro-optical systems;
- Choosing appropriate gyroscope for video stabilization;
- Integration of video stabilization in the long-range electro-optical systems;
- Algorithm improvements for gyroscope noise suppression.

The paper is organized as follows. In Section 2, we describe each step of the proposed stabilization method in detail. Section 3 discusses the most important characteristics of MEMS gyroscopes for the stabilization applications and the gyroscope selection criteria. Section 4 describes our implementation of the proposed algorithm and presents the hardware platform together with a discussion on practical stabilization improvements for the problems of gyro bias instability, noise and black boarder (cropping) problem. In Section 5, we present the experimental results with a performance comparison with Google and RFEL feature-based digital stabilization algorithms as well as advantages and disadvantages of the proposed stabilization. Finally, the conclusion is provided in Section 6.

## 2. Algorithm Description

The general algorithm is described in paper [10] and here we present the necessary theoretical background and needed adjustment for its implementation into the long-range electro-optical systems. We start with the camera motion model in 3D space where gyroscope measurements are obtained, then explain the proposed unwanted motion estimation (filtering) process, and finish with the transformation in the 2D image plane for final homographic video stabilization. The method for calibration of the camera and sensor parameters is then presented, including time synchronization of the gyroscope samples and the video frames. 

### 2.1. Camera Motion Model

**Figure 3:**
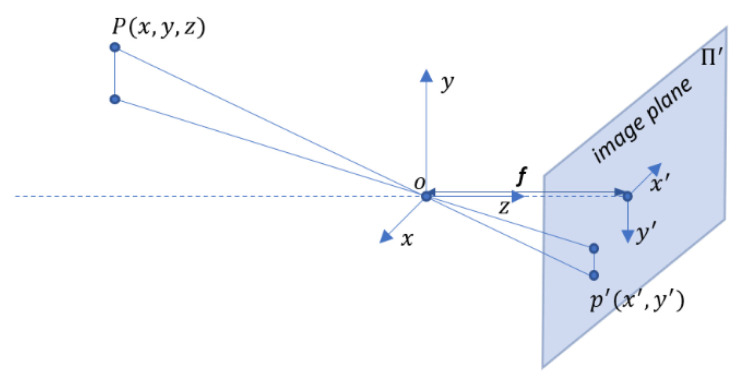
Pinhole camera model.

First, we need to establish a one-to-one mapping between points in the 3D camera coordinate frame and points on the image plane. The model of the camera movement is based on the pinhole camera model (Figure 3).

Let $P={\left[x,y,z\right]}^{T}$ be a point on some 3D object visible to the pinhole camera. *P* will be mapped or projected onto the image plane $\Pi^{\prime }$, resulting in a point $p^{\prime }=\left[x^{\prime },y^{\prime }\right]$. Relationship between homogenous coordinates of point $P_{h}={\left[x,y,z,\;1\right]}^{T}$, in 3D global coordinate frame, and ${p_{h}}^{\prime }=\left[x^{\prime },y^{\prime },\;1\right]$, in 2D image plane, is:

(1) $${p_{h}}^{\prime }=K\left[I\;0\right]P_{h}.$$

A detailed explanation of this relationship can be found in [19]. K is the camera intrinsic matrix that contains critical parameters needed to characterize the camera model:

(2) $$K=\left[\begin{array}{ccc} f & 0 & o_{x}\\ 0 & f & o_{y}\\ 0 & 0 & 1 \end{array}\right],$$

where $\left[o_{x},o_{y}\right]$ is the origin of the camera axis in the image plane (ideally $o_{x}=o_{y}=0$) and f is the focal length. These parameters are generally unknown (or known inaccurately) and should be determined in the camera calibration process. Here, it has been assumed that the camera has square pixels (square sensor). Generalizing to possible rectangular pixels is easy (e.g., [14]). Information about the focal length can be determined from FOV. We know the exact value of FOV (horizontal or vertical) from the lenses.

Camera orientation movement is described with the rotation matrix R(t) at time t, which can be calculated from Euler angles obtained by integration of the angular velocities measured by the gyroscope. Hence, for each 3D point P we have, the corresponding 2D point on the recorded image p is:

(3) p=KR(t)P.

The change of angle from time $t_{1}$ to time $t_{2}$ is obtained from gyroscope measurements integration as follows:

(4) $$\theta \left(t_{2}\right)-\theta \left(t_{1}\right)=\overset{t_{2}}{\underset{t_{s}=t_{1}\;}{\sum }}\tilde{\omega }\left(t_{s}+t_{d}\right)\Delta t$$

where Δt is the sampling time of gyroscope measurements (it is assumed that we sample the gyroscope faster than the video sequence, e.g., framerate is 30 Hz and gyroscope sample frequency is 500 Hz), $\tilde{\omega }\left(t_{s}+t_{d}\right)$ is measured angular velocity, which is in general shifted by $t_{d}$ relative to the moment of frame capture (due to different unknown delays in hardware and software processing of different sensor’s readings), and it is given by:

(5) $$\tilde{\omega }\left(t_{s}+t_{d}\right)=\omega \left(t_{s}+t_{d}\right)+\omega^{d}+\xi \left(t_{s}+t_{d}\right)$$

where $\omega^{d}$ is the gyroscope drift and $\xi \left(t_{s}+t_{d}\right)$ is a stochastic measurement noise with zero mean. Here, it is assumed that gyroscope axes ideally match with axes of camera coordinate system. If this is not the case, in Equation (3), the permutation matrix T, which represents the transformation between two coordinate systems, is added:

(6) p=KTR(t)P

Therefore, the additional unknown parameters to be determined in the calibration process are: time delay $t_{d}$, gyroscope drift $\omega^{d}$ and permutation matrix T.

### 2.2. Estimation (Filtering) of Unwanted Camera Movement

When the rotation matrix (camera orientation) R(t) has been determined as described in the previous section, the next step is to filter out the wanted motion which is, in the considered surveillance applications, typically in the low frequency spectrum. For a more compact notation and simpler mathematical analysis, 3D rotations are usually represented by unit quaternions instead of matrices. Hence, in this chapter, we denote the orientation of the camera (with the standard matrix notation R(t)) using the quaternion q(t).

Spherical linear interpolation (SLERP) method was first proposed in [20] in the context of interpolation of unit quaternions and represents a generalization of a standard linear interpolation (LERP) between two vectors a and b, LERP(a,b,α)=(1−α)a+αb, 0≤α ≤1, to the case when the interpolation is performed according to the spherical geodesic distances. In the context of filtering rotational quaternions (camera orientation), the SLERP method is used as follows:

(7) $$q_{f}\left(t\right)=\mathrm{SLERP}\left(q_{f}\left(t-1\right),q\left(t\right),\alpha \right),$$

where $q_{f}\left(t\right)$ is the filtered orientation, q(t) is the measured orientation at time t, and α is the interpolation parameter. It can be shown that the SLERP method is equivalent to the first-order IIR filtering in Euclidean space, with a spherically geodesic metric used instead of the standard Euclidean metric [9].

### 2.3. Image Transformation

**Figure 4:**
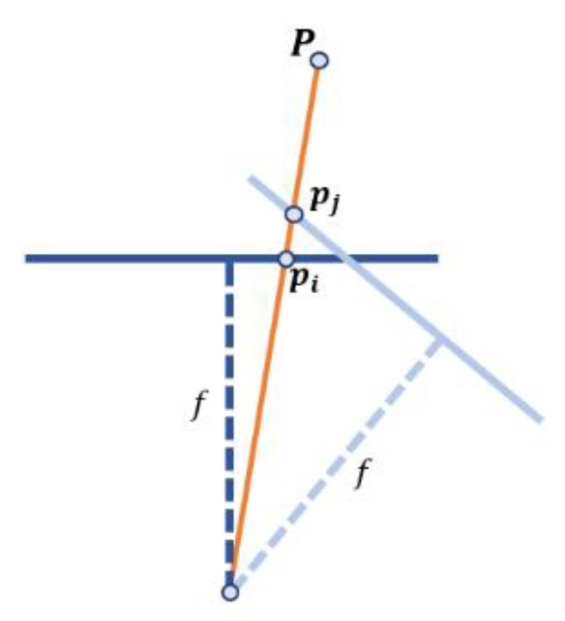
Two orientations of the camera and the corresponding images *i* and *j*.

Now, we derive a connection between the corresponding points in two adjacent frames corresponding to the two camera orientations (Figure 4).

For a given point P in the scene, the projected points $p_{i}$ and $p_{j}$ in the image plane for two frames i and j at the moments $t_{i}$ and $t_{j}$ are given as follows (see (6)):

(8) $$p_{i}=KTR\left(t_{i}\right)P,\;\;p_{j}=KTR\left(t_{j}\right)P$$

By removing the variable P from the above equations, we obtain a mapping between the points of the frame i and frame j:

(9) $$p_{j}=W\left(t_{j},t_{i}\right)p_{i}$$

where $W\left(t_{j},t_{i}\right)=KTR\left(t_{i}\right)R{\left(t_{j}\right)}^{T}T^{-1}K^{-1}$ is the warp matrix that defines the transformation (homography), transforming each point in the frame j to the corresponding points of the previous frame i. The two most significant values from warp matrix are elements that represent translation by *x* and *y* axes.

### 2.4. Offline Calibration

In the previous section, we have seen that the final transformation (9) depends on several unknown parameters: coordinate system transformation T, time shift between gyroscope and camera measurements $t_{d}$, gyroscope offset $\omega^{d}$ and camera axis coordinates $\left[o_{x},o_{y}\right].$ Hence, the quality of the video stabilization will directly depend on how precisely we know these parameters. Certain parameters may be specified by the manufacturer; however, for the purpose of greater precision, prior to the commissioning of the algorithm defined above, it is necessary to perform a calibration in order to accurately determine the above parameters. Calibration is performed offline on a recorded video sequence and measurements from the gyroscope. Here, we describe the calibration process used in [10,14]. It can be performed by recording a short video clip of the scene on which the feature-based stabilization method is based only on video processing, which can give almost ideal results (e.g., [2]), together with the corresponding gyroscope measurements. The scene should have sharply defined features and should be without wanted motion without object motions on the recorded scene. The system should be immobile at the beginning and after the shake. By applying a method for detecting and tracking frame-to-frame features (e.g., SIFT method [21]), we obtain a set of corresponding point pairs ($p_{i}$, $p_{j}$) for all adjacent frames in the recorded video. On this basis, an optimization problem is formulated, where we want to find the parameters of the matrix W from (9) that minimize the mean square error

(10) $$J=\underset{\left(i,j\right)}{\sum }{\left|\left|p_{j}-W\left(t_{j},t_{i}\right)p_{i}\right|\right|}^{2}$$

where the sum is made over all pairs of points ($p_{i}$, $p_{j}$) and all pairs of recorded frames. This is a nonlinear optimization problem that we solve using standard methods [10,14].

## 3. Choosing Right Gyroscope for Video Stabilization

For a concrete practical application based on the above method description, it is required to choose the appropriate gyroscope. The quality of video stabilization largely depends on the quality of sensor. Compromise between the price and quality must be achieved. For instance, high quality 3-axis fiber optic gyroscope (FOG) costs about $10,000 and it provides superior performances. When properly used, an insignificant performance decrease may be achieved by using lower cost MEMS gyroscopes, which target price is about $10. In this section, we described the sensor selection criteria and the key sensor features that should be considered. 

We restrict our choice to MEMS sensor because they are low cost, small and lightweight compared to the other available technologies. In addition, performance of the MEMS gyroscope is improving rapidly. 

### 3.1. Theoretical Background

Before we present a method of choosing a gyroscope, we give a necessary theoretical background. When choosing a specific MEMS gyroscope for video stabilization, it is necessary to consider the following performance factors as shown in [22]: bias instability, angle random walk (ARW), temperature sensitivity and quantization error.

#### 3.1.1. Bias Instability

When the input rotation is null, the output of gyroscope may be nonzero; the measured value is called bias offset. In the previous section in Equation (5), we assumed that bias offset is constant when measuring angular velocity and that it can be determined in the offline calibration process. However, in practice, this bias will change slightly over the long term due to the changes in the environmental conditions. It causes drift in angular orientation obtained by integration of the angular velocity measurements; hence, the term gyroscope drift is often used for bias instability. A bias instability describes how the bias of a device may change over a specified period of time at constant temperature. It is usually expressed in unit °/h or °/s for less accurate devices. It can be measured using the Allan Variance technique [23]. Its influence is greater on longer measurement periods; thus, it is one of the most critical factors in the gyroscope selection process. The measurements error of frame-to-frame orientation change due to long-term bias instability can be calculated using the formula:

(11) $$E_{drift}\approx BI\frac{1}{f_{R}}$$

where *BI* is bias instability expressed in °/s and $f_{R}$ is frame rate.

The consequence of bias instability on a stabilized image is the occurrence of image drift. In Section 4.2.1, we will show how the problem of bias instability is solved. 

#### 3.1.2. Angle Random Walk

In the output of a gyro, there is always a broadband white noise element. We are interested in how this noise affects the integrated signal. Standard deviation of the integrated signal grows proportionally to the square root of time under the influence of white nose [24]. In the manufacturer’s specifications, this noise is listed as angle random walk (ARW) or rate noise density (RND) in ${}^{\circ}/s/\sqrt{\mathrm{Hz}}$ or ${}^{\circ}/\sqrt{s}$. ARW [25] is a noise specification that is directly applicable to angle calculation. It describes an average deviation or an error that occurs when the integration of the measurements is performed. ARW can also be specified using the Allan Variance technique.

Standard deviation of the error (in degrees) due to this noise, when calculating the angular movement from frame to frame, can be calculated by:

(12) $$E_{std}\approx RND\times \sqrt{BW}\frac{1}{f_{R}}$$

where *RND* is the noise intensity density specified by the manufacturer, $f_{R}$ is frame rate of videos sequence, *BW* is the frequency bandwidth of gyroscope signal (maximum is $\frac{f_{R}}{2}$).

In video stabilization, ARW is manifested by the occurrence of video shaking, including when the original video is completely stable. Hence, the influence of ARW on stabilization is noticeable when the camera system is immobile, and it is more pronounced when the field of view is narrower. When the unwanted motion that needs to be stabilized is present, image shaking due to noise is imperceptible (since the camera movements are more significant). In Section 4.2.2, we present a solution to the problem of noise-based stabilized video shaking.

#### 3.1.3. Temperature Sensitivity

The gyroscope performance changes with the operating temperature. Temperature fluctuations due to the changes in the environment, and sensor self-heating induces movement of the gyro bias. The relationship between the bias and temperature is often highly nonlinear. To correct the effect of temperature changes on the bias, it is necessary for a gyro device to contain internal temperature sensors.

#### 3.1.4. Quantization Error

The quantization error is typically referred to as gyroscope sensitivity in manufacturer specification and it is expressed in (°/s)/LSB. Therefore, the measurement error of changing the orientation of the camera from frame-to-frame due to quantization error can be calculated by the formula:

(13) $$E_{q}\approx SENS\;\frac{1}{f_{R}}$$

where SENS is sensitivity which is desirable to be as low as possible and $f_{R}$ is frame rate. The smaller the measuring range, the lower the sensitivity. The measurement range of most commercially available MEMS gyroscopes is from ±250°/s to ±2000°/s.

### 3.2. Gyroscope Selection

Error due to the long-term bias instability is the dominant cause of measurement errors of the camera orientation. This error can be drastically reduced if we estimate bias value in real time. Estimation can be performed using a fusion of gyroscope measurements with accelerometer and/or magnetometer measurements. By using only gyroscope and accelerometer measurements, it is possible to estimate pitch and roll [26]. For yaw estimation, we also need a magnetometer [27]. Hence, if we use a complete IMU device instead of only a gyroscope, we can, to a certain extent, eliminate error due to bias instability. The reason why we have decided for the gyroscope-only approach is the impact of other devices on magnetometer measurements. Sensors are placed into the camera housing where there are also devices such as a compressor for thermal camera, heater, blower, power supply, etc. In such an environment, the magnetometer gathers considerable noise.

**Figure 5:**
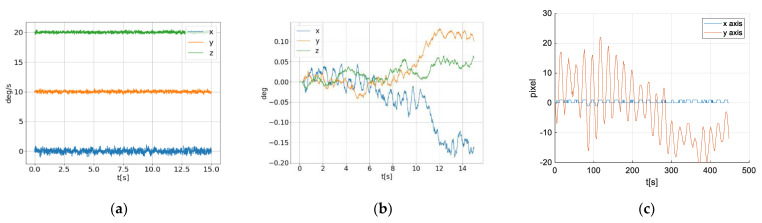
Measurements when FOV is narrow (1.94 deg—vertical FOV). (**a**) Gyroscope measurements—angular velocity. *y* and *z* axis are offset such that all signals are visible. (**b**) Angle calculated by integration of the gyroscope measurements. (**c**) Orientation movements in the image plane, calculated from the recorded video.

We considered several gyroscopes such as L3GD20H [28], I3G2450D [29], BNO055 [30], L3G3IS [31], FXAS21002 [32], ICG20330 [33]. Based on the above considerations, we analyzed all mentioned devices and compared sensor characteristics from manufacturers specifications and concluded that all sensors, except ICG20330, have similar parameters. We initially decided to use gyroscope FXAS21002 because we found that the solution integrated on a printed circuit board (PCB) is the best choice for testing. We used an IMU device [34], which contains an accelerometer and magnetometer beside the gyroscope. Full-scale range is set to the narrowest range ±250°/s. For such systems, expected angular velocities caused by outside disturbances are small, up to several °/s. In a laboratory environment, when the field of view is large, we obtained a satisfying result. However, in the field condition, when FOV is significantly lower, the amplitude of useful signal is the same as the amplitude of noise, see Figure 5a. In that record, disturbances (wind) caused the system to move along only one axis (*x* axis of gyroscope, which is mapped to the *y* axis in the image).

**Figure 6:**
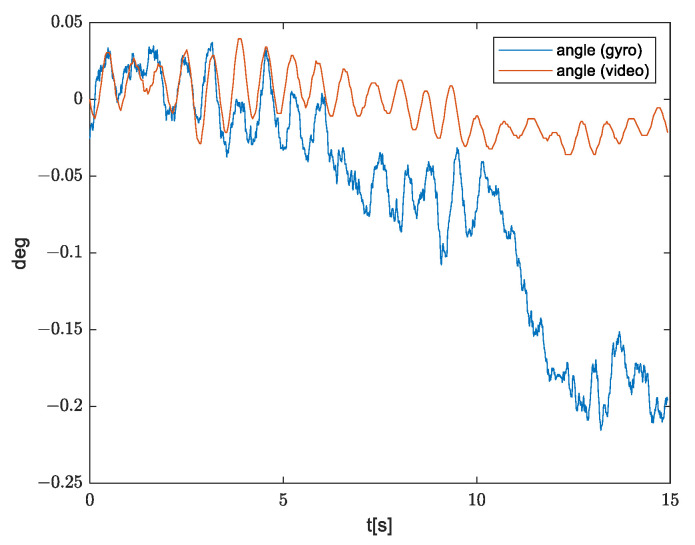
Movements calculated by integration of gyroscope measurement (blue) and movements calculated based on recorded video (orange).

In the signals shown in Figure 5b, it should be expected to observe more oscillations along *x* axis angle, but compared to the signals from the other two axes, the amplitude of these oscillations is similar to the error due to ARW. In order to prove this, we calculated movements based on a recorded video (Figure 5c). Movements are expressed in pixels, but knowing FOV, it is easy to convert these values into degrees. Figure 6 shows angular movements obtained by integration of gyroscope measurements and from the video record, where it can be observed that ARW is more dominant compared to the measured oscillations.

**Figure 7:**
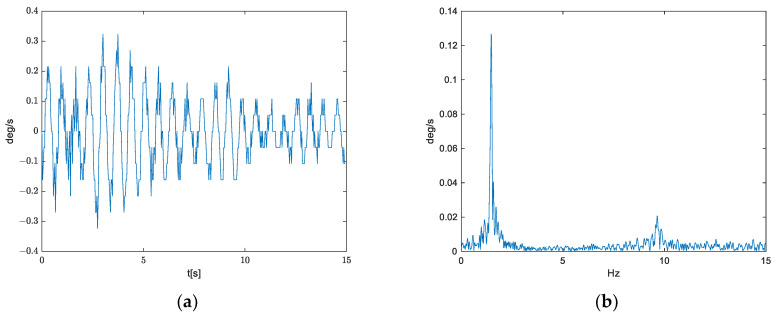
(**a**) Angular velocity and (**b**) amplitude spectrum, obtained by differentiating movements calculated on the basis of the video record.

By differentiation of the movements calculated on the basis of the video record, we have obtained properties of angular velocity of which the gyroscope needs to satisfy. Here, we have used the small-angle approximation. The obtained signal is shown in Figure 7. 

From Figure 7, we can notice that the useful signal is mostly in the range from −0.3°/s to 0.3°/s. 16-bit number is used for full-scale range ±250°/s, such that the useful signal is presented using at most 6 bits. 

**Figure 8:**
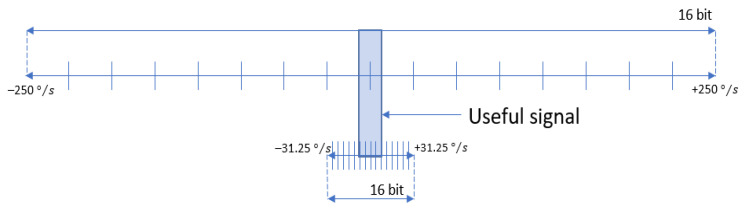
Different full-scale ranges and the useful signal range.

We can conclude that with this sensor it is not possible to stabilize the image when the FOV is narrow. We needed a sensor with a narrower range (better sensitivity) and lower ARW. Based on the requirements of our long-range surveillance applications, we selected to use ICG20330 sensor, whose range is the narrowest, from ±31.25 to ±250°/s, and ARW is the smallest. Figure 8 shows the ratio of the full-scale ranges, ±31.25 and ±250°/s, to the useful signal range; hence, ±31.25°/s range was selected.

In this paper, we used only the ICG20330, but if another sensor with similar or better sensitivity is used, the desired result can be obtained. In addition, if a sensor with significantly worse sensitivity is used, the proposed method will fail to stabilize small disturbances that are noticeable when FOV is narrow. This is a limitation that depends on the gyroscope.

## 4. Practical Implementation

The sensor and video signal processing module (vVSP) are placed in the camera housing. The gyroscope is mechanically coupled to the camera, such that the measurements correspond to the movements of the camera. The inputs to the vVSP module are a sequence of the recorded video frames and the corresponding gyroscope samples. The interface board is responsible for video signal conversion. Video frames and gyroscope measurements are captured, pre-processed, synchronized and forwarded for further processing in the FPGA module Xilinx KINTEX-7 [35]. The FPGA module implements MicroBlaze [36] microcontroller that reads measurements from the gyroscope via i2c bus. The gyroscope sends the interrupt signal when data is ready. Based on the synchronization signal that indicates the beginning of a new frame (FrameValid), microcontroller stores measurements in the corresponding frame. Measurements are embedded into the first line of frame, and this is imperceptible to the end user. 

**Figure 9:**
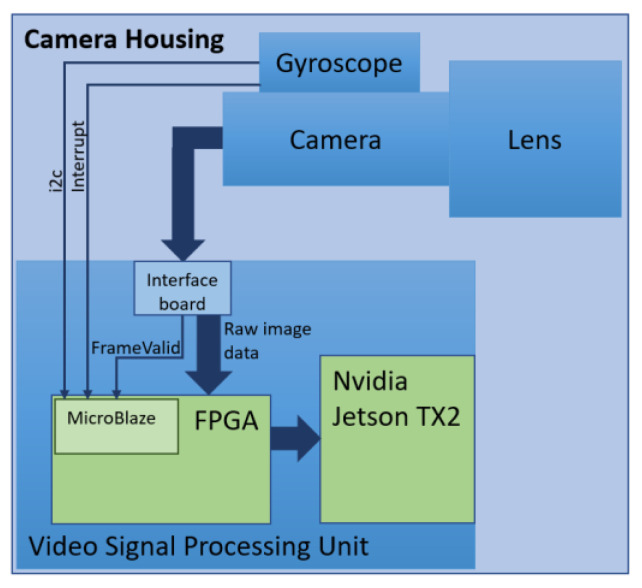
Camera housing—block diagram.

**Figure 10:**
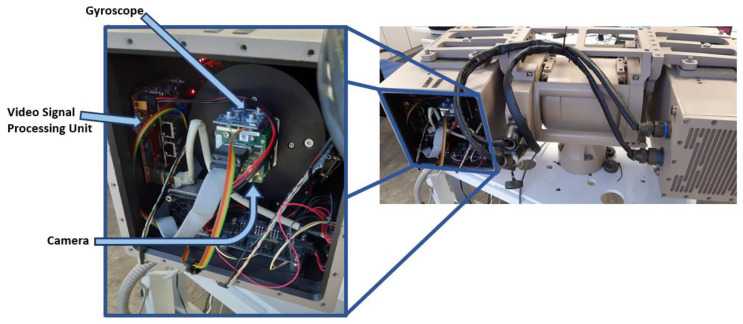
Electro-optical system (**right**) with opened camera housing (**left**) to show hardware required for the gyroscope-based stabilization.

The described stabilization algorithm is executed on Nvidia Jetson TX2 [37] module. Jetson TX2 is based on the quad-core ARM processor and 256 CUDA graphical core. The algorithm is implemented in C++ as a Gstreamer [38] plugin. Image warping is implemented using Nvidia VisionWorks [39] toolkit and it is executed on CUDA core. Figure 9 illustrates data flow from sensors to the Jetson TX2 where the video is being stabilized. In Figure 10, we can see how it physically appears in the electro-optical system.

### 4.1. Algorithm Flow

**Figure 11:**
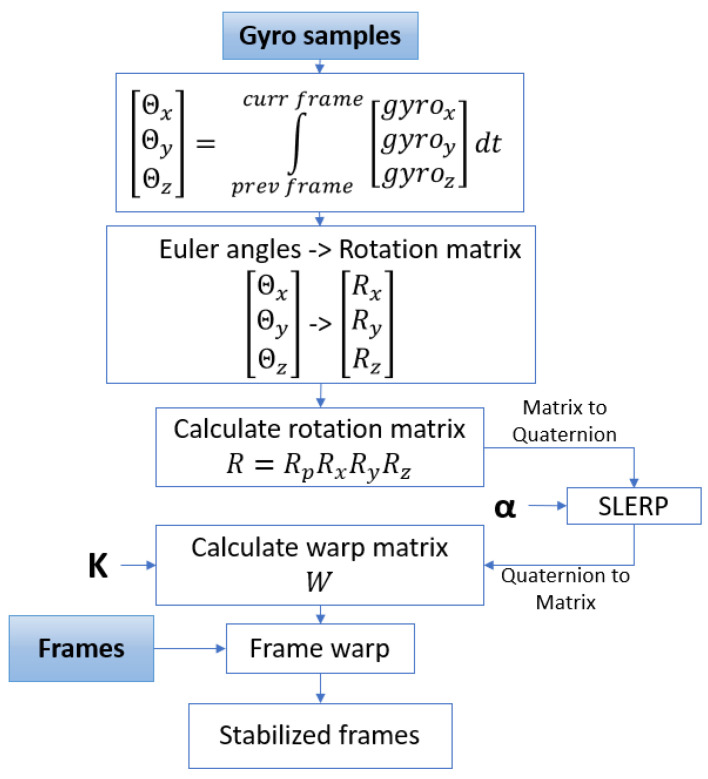
Stabilization algorithm—block diagram.

According to the algorithm description given in Section 2, gyroscope samples are integrated in order to obtain camera orientation expressed in Euler angles. The integration period is from the previous frame to the current frame. Calculated angles are transformed to the corresponding rotation matrices. The new rotation matrix R is calculated based on the rotation matrices $R_{x},\;R_{y},\;R_{z}$ and rotation matrix from previous frame $R_{p}$. The calculated rotation matrix is transformed to the quaternion and the SLERP function is applied in order to filter camera motion. The input parameter to SLERP function is the interpolation parameter α. In Section 4.2.1, it is explained how the parameter α is determined. After the filtration in the quaternion space, we return to the domain of rotation matrices. This is where the stabilizing warp matrix is calculated using camera intrinsic matrix K, the rotation matrix and the parameters obtained by calibration, explained in Section 2.4. The last step is image warping after which we obtain a stabilized image corresponding to the current frame. A block diagram of the described algorithm is shown in Figure 11.

### 4.2. Algorithm Extensions and Practical Improvements

In this subsection, we present our solutions to the problems caused by gyroscope bias instability and ARW discussed in Section 3.1. In addition, this section covers a solution to the black border problem, which appears after image warping when the black parts appear in the stabilized image such that cropping is necessary. 

#### 4.2.1. Solution for Bias Instability

**Figure 12:**
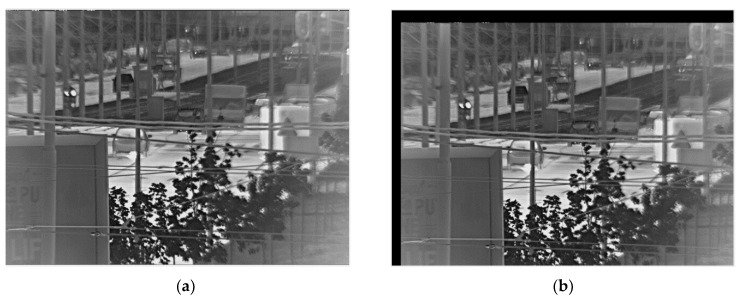
Influence of bias instability. (**a**) Stabilized image at the beginning of video; (**b**) image after a few minutes.

A consequence of bias instability on the stabilized image is the constant movement of the image inside frame, when the black border appears and grows over time. This is illustrated in the Figure 12.

To remove negative effect of gyroscope bias instability, it is necessary to move the position of reference frame together with the gyroscope drift. The reference frame has been unchanged thus far, and it is determined by position of the frame when stabilization has started. If the reference frame were moving with the drift, the observed object would always be in the same position. However, the question is how to move the reference frame together with the drift and to avoid reference frame moving with the oscillations caused by disturbances.

**Figure 13:**
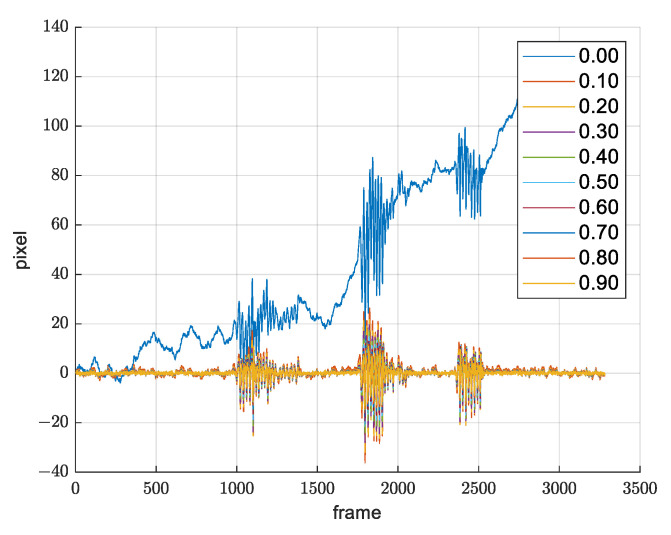
SLERP parameter α selection.

Besides trajectory smoothing, an additional advantage of using SLERP filtering, explained in Section 2.2, is slowly changing drift suppression. By selecting the α parameter correctly, it is possible to achieve the desirable moving of the reference frame. Figure 13 shows that, for each value of the α parameter greater than zero, the drift will be suppressed.

**Figure 14:**
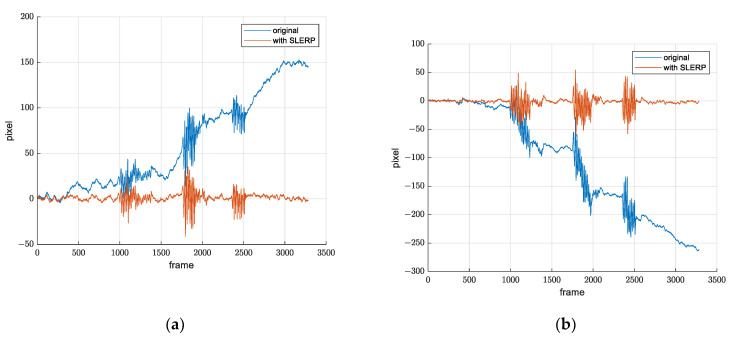
Demonstration of drift suppression. Signal before and after SLERP. (**a**) *x* axis translation from warp matrix; (**b**) *y* axis translation from warp matrix.

It was experimentally determined that, for the value α=0.03, we obtain the desirable movement of the reference frame and desirable movement filtration, which means that the resulting quaternion $q_{f}\left(t\right)$ takes 3% of $q_{f}\left(t-1\right)$ and 97% of q(t), which is sufficient for filtering out the image movements happening due to the slow drift changes. Figure 14 shows *x*-axis and *y*-axis translation from warp matrix before and after applying SLERP. 

**Figure 15:**
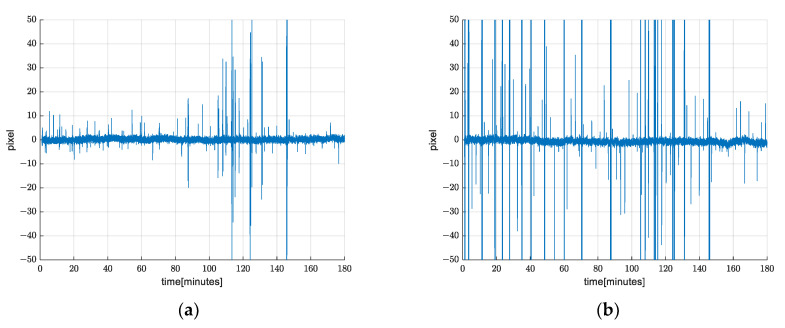
Demonstration of drift suppression over a longer time period. (**a**) *x* axis translation from warp matrix; (**b**) *y* axis translation from warp matrix.

Since the drift grows over time, it is necessary to observe how this method performs over a longer time period. A three-hour record was created, and translation from warp matrices is shown in Figure 15. We see that the drift is not a problem, including after a few hours. Mean value is approximately zero throughout the whole record.

#### 4.2.2. Removing the Artifact Noise-Based Video Oscillations 

In a situation without outside disturbances (camera shaking), when the original video is stable, without unwanted motion, application of the above stabilization method results in a video with undesirable slight image shakings. As discussed in Section 3.1.2, this is expected due to the presence of ARW from the gyroscope measurements. This is an unacceptable effect for the end user since the stabilization needs to be turned on independently of the presence of disturbances. The human operator expects to have a stabilized and clear image in every situation. Hence, we need to design a method eliminating the ARW-based oscillation in the state without the presence of disturbances. Since these noise artifacts are in the same frequency band as the typical real camera disturbances, simple low pass filtering cannot be applied.

We propose to use the short-time variance (STV) estimate, using a rectangular window and compare it against a threshold in order to estimate a time instance in which the stabilization should be automatically enabled or disabled. 

**Figure 16:**
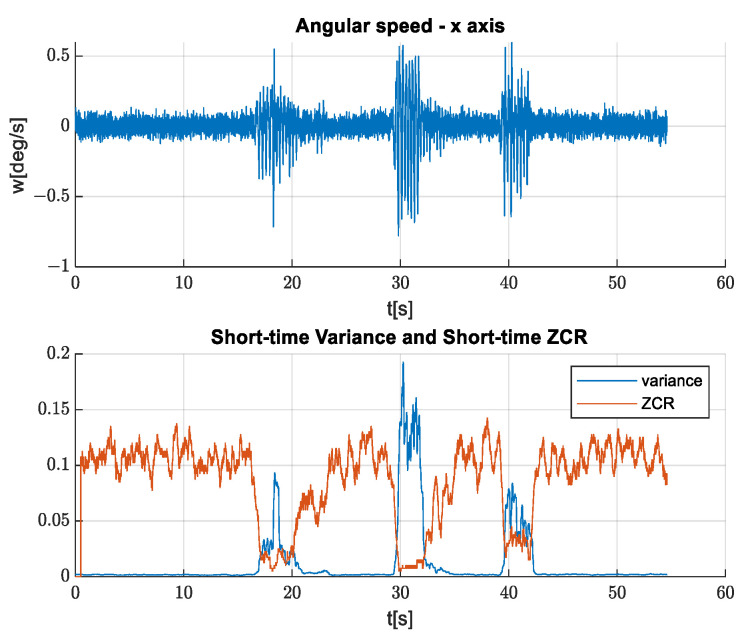
Short-time variance and short-time ZCR.

In Figure 16, an example of the STV estimate is depicted together with the zero-crossing rate (ZCR). It can be concluded that the STV is better to be used for stabilization auto-enabling since ZCR is related to the disturbance frequency. 

**Figure 17:**
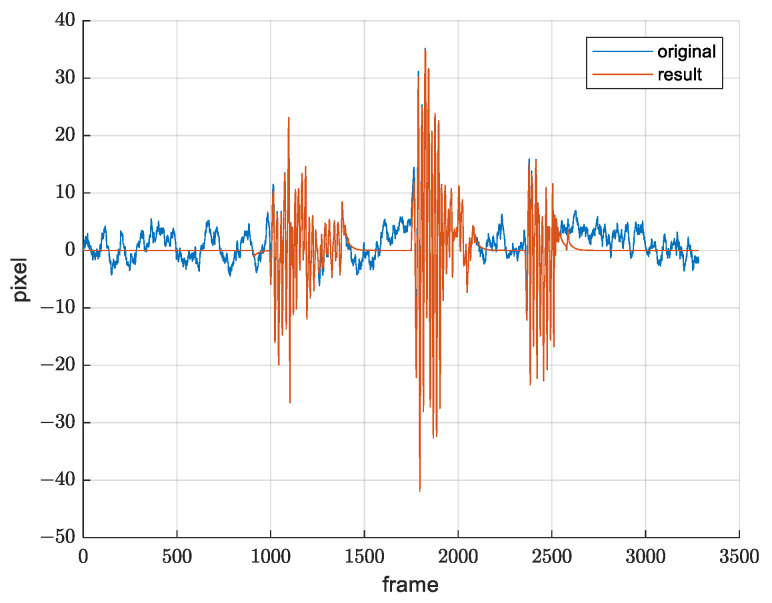
*x* axis translation obtained from the warp matrix. Comparison of the original record and the result of elimination of the noise-based artifact oscillations.

This solution is implemented by calculating the STV in a window with the length of *n* samples (in this case *n =* 50). The threshold above which the video stabilization should be auto-enabled is experimentally determined. The result is shown in Figure 17. 

#### 4.2.3. Adaptive Crop—Black Border Problem

As a result of image warping, stabilized frames typically contain black areas near the frame borders. These areas can be eliminated by cropping. One simple solution is to set the crop factor to be larger than the maximum expected image movement. We can determine this value from the warp matrix by calculating translation along *x* axis and *y* axis. A drawback of this solution is that typically more image content than needed is removed in order to avoid black borders in extreme situations. 

**Figure 18:**
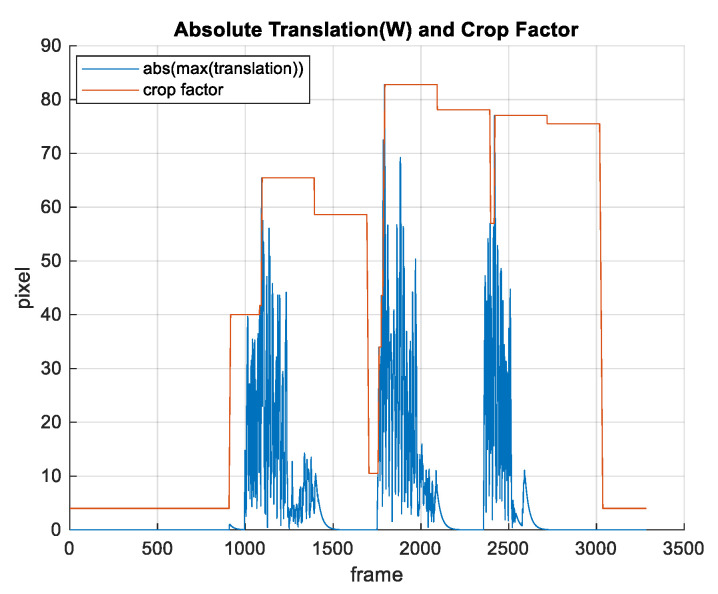
Crop factor assessment.

Our solution is to adaptively change the crop factor based on the warp matrix (translation along *x* and *y* axis). The idea is to change the crop factor every *n* seconds based on the maximum translation obtained from the warp matrices in the last *n* second, except in situations when the translation is larger than the current crop factor. If this happens, the crop factor is changed immediately, and this value remains unchanged for the next *n* seconds. Cropping is performed such that the aspect ratio is preserved. The change of the crop factor is performed progressively (maximum 4 pixels per frame) in order to obtain a smooth video without abrupt changes. It sometimes may appear to be a zoom change. Figure 18 illustrates the assessment of the crop factor based on warp matrix.

## 5. Experimental Results and Discussion

**Figure 19:**
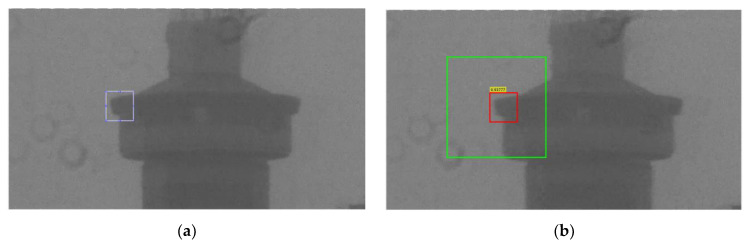
An illustrative presentation of the method for determining the quality of stabilization. (**a**) Selecting object on the first frame. (**b**) A frame after the stabilization is turned on. The green box represents the region where the tracking algorithm is searching for the marked object. The red box represents the region where the object is found. In addition, the given numeric value tells us how sure the algorithm is that the detected object is the one we are looking for.

In order to graphically present the quality of stabilization, the amount of unwanted movements of an object on the image should be measured. For this purpose, we have used a normalized two-dimensional cross-correlation function. We have marked one object that appears on each frame and tracked its movement from frame to frame. The method searches for the position of the marked object in the region where we expect that marked object to appear after the movement. We assume that the tracking algorithm gives us the true position of the tracked object. As a result, we obtain displacement of the object along the x and y axes. An illustrative representation of this method is shown in Figure 19.

**Figure 20:**
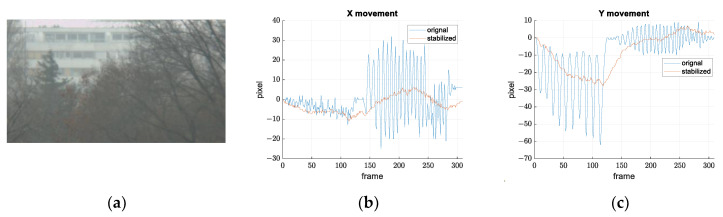
Results of our video stabilization and comparison with the original video. (**a**) A frame from the video that is stabilized. (**b**) Movement of a selected object along x axis. (**c**) Movement of a selected object along y axis.

We performed the described procedure on the original and the stabilized video. The result is shown in the Figure 20. Later, we used same method to compare the other two approaches. 

On the presented graphs, we can see that our approach has stabilized the video successfully. The remaining oscillations (amplitude of a few pixels) that can be noticed are a consequence of sensor imperfections discussed earlier, mostly due to the additive noise. In order to rate the quality of our stabilization, we compared it with the stabilization from other manufacturers.

### 5.1. Stabilization Comparison Results 

We have compared our solution with RFEL and Google (Google Photos [40]) digital feature-based stabilization methods. RFEL is applied in real time, while Google is offline. 

RFEL RPDS [41] platform is a flexible real-time video processing platform with the primary processing function of digital stabilization. It supports Base CameraLink and HD-SDI input. RFEL stabilization is capable of stabilizing up to 50 pixels frame-to-frame translation and up to 5 degrees frame-to-frame rotations. 

We have made a stabilization comparison on several representative recordings. It is expected that feature-based stabilizations work well on videos where the objects appear with noticeable edges because it is easier to find and track the features. However, it is important to compare these stabilization methods in non-ideal conditions:

- scenes taken in bad weather such as rain, snow, fog, haze, dust;
- when the image is not focused (blurred);
- when there are no noticeable edges or shapes suitable for features extraction (sea, desert, forest, sky);
- when there are a lot of moving objects;
- with some distortions such as stains on the camera glass.

The Google algorithm adaptively changes digital zoom (FOV) depending on amplitude of the disturbance. As a result, position of the tracked object is not in the same place such as in the original video. It can cause a noticeable offset between object position in Google stabilization and other stabilizations. We will ignore it because we are only interested in the shape of the curve, i.e., smoothness of the curve.

**Figure 21:**
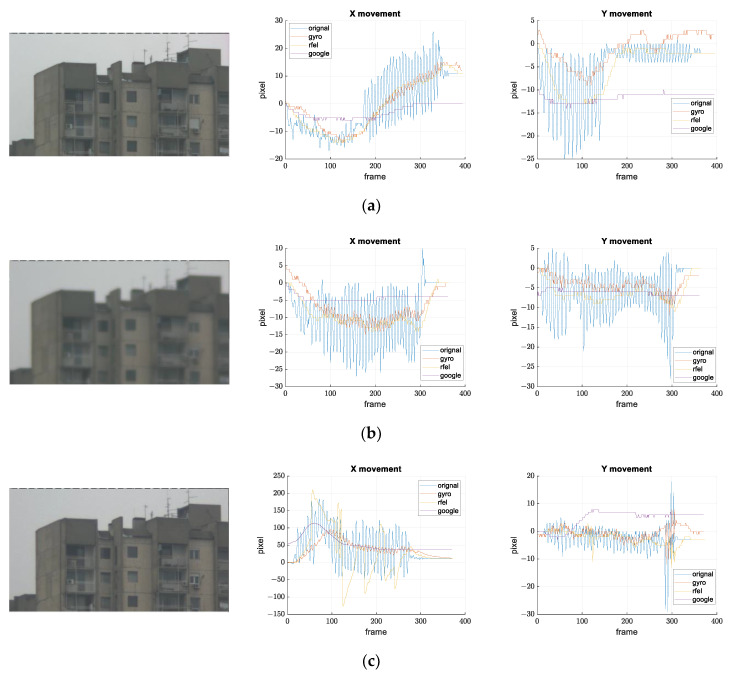
Results of the stabilization comparison on video records suitable for feature-based digital stabilization. Camera looks at the building with many noticeable edges (shapes). Frames from the recorded video and the movements on the image plane along *x* and *y* axes are shown for all the compared methods for: (**a**) small disturbance amplitude, (**b**) small disturbance amplitude, blurred and out-of-focus image; (**c**) large disturbance amplitude.

**Figure 22:**
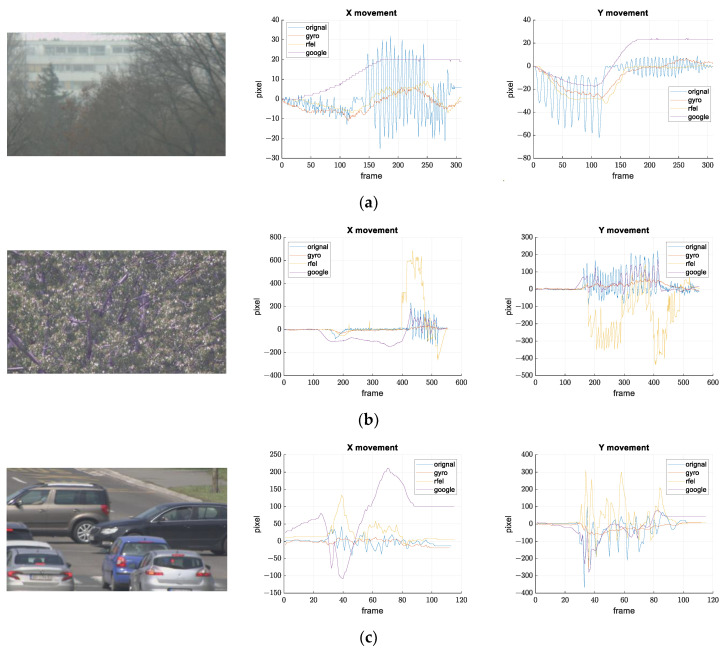
Results of the stabilization comparison on video records are difficult for features extraction and tracking. Frames from the recorded video and the movements on the image plane along *x* and *y* axes are shown for all the compared methods: (**a**) camera looks at the tree canopy and a building in the background; (**b**) camera looks only at the tree canopy; (**c**) camera looks at crossroad with many moving objects (cars).

**Figure 23:**
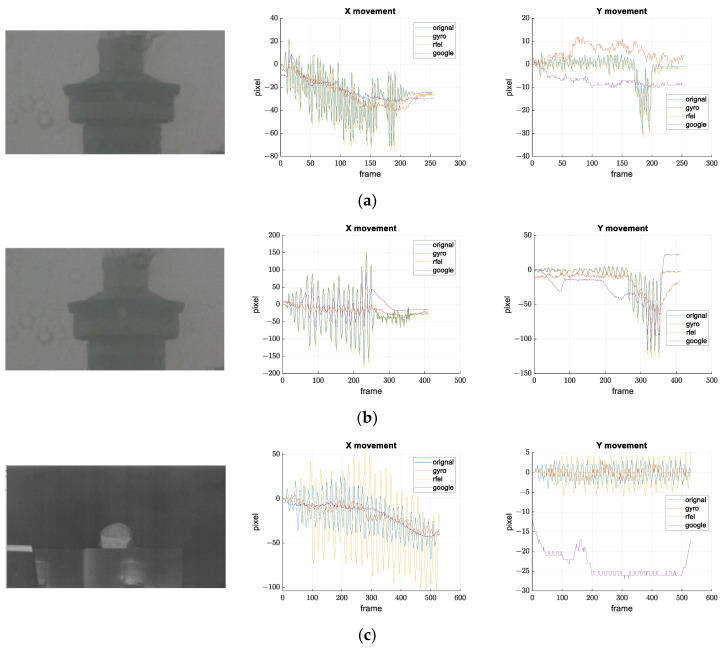
Results of the stabilization comparison on video records with stains on camera glass and a video record from a thermal camera. Frames from the recorded video and the movements on the image plane along *x* and *y* axes are shown for all the compared methods for: (**a**) small disturbance amplitude and stains on the camera glass, (**b**) larger disturbance amplitude, and stains on the camera glass (**c**) thermal camera.

Figure 21, Figure 22 and Figure 23 show the results of the comparison.

In examples shown in Figure 21, the Google method shows the best results, which is to be expected because it is offline stabilization, and the conditions are acceptable for feature-based methods. Ours and the RFEL stabilization have similar results, except on the record (c), where RFEL fails to stabilize larger disturbances.

Records shown in Figure 22 should simulate scenes where feature extraction and tracking are difficult. Record (a) shows that digital stabilizations work well if objects (building in the background) with clearly expressed features are presented; in addition, the tree canopy is dominant in the scene. If there are no such objects, such as in record (b), only our gyroscope stabilization successfully stabilized the image. Record (c) represents scenes with many moving objects. This is another example where feature extraction and frame-to-frame tracking is difficult or impossible. In addition, this is one more example where gyroscope stabilization shows its advantages. 

Maintenance and cleaning of the surveillance systems are rare since the system is often difficult to access. Because of this, stains can sometimes be seen on the camera glass, which can cause that feature-based stabilization algorithms detect and track features from the stains. Stains are easier to spot when the FOV is narrower. That is exactly what happened on records (a) and (b) in Figure 23. Google manages to stabilize images when the amplitude of the oscillation is small, while RFEL stabilization follows only features from stains on both records. Results from Figure 23c show that certain digital stabilizations do not manage with an image that is not in visible range. These records also show advantages of our solution.

### 5.2. Execution Time

**Figure 24:**
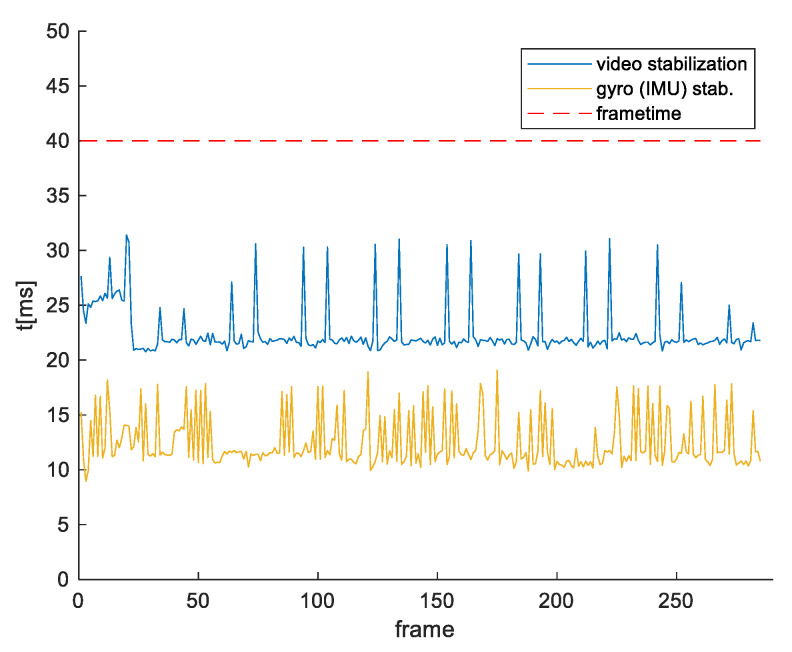
Time execution on the same hardware platform of our gyroscope-based method and feature-based method.

It is also important to compare the execution times of our stabilization algorithm and the feature-based stabilization. Both stabilizations are executed on the same platform, Jetson TX2, and on the same video record. For this purpose, we used the feature-based stabilization described in [42], which is also executed using CUDA core and NVIDA VisionWorks toolkit. Results are shown in Figure 24.

### 5.3. Advantages and Drawbacks of the Proposed Method

Based on the presented results, we here report the advantages and disadvantages of our gyroscope-based video stabilization compared to the other digital stabilization methods. 

Advantages:

- Price. The price of a fiber optic gyroscope (FOG) is about $3000 per axis of rotation. The price of an RFEL stabilization device is higher than for one-axis FOG. Price of MEMS gyroscope is only about $10 per camera;
- Drastically lower computational complexity. The only operations that need to be performed are integration of the gyroscope samples, 3D matrix multiplications and image warping. Compared to the stabilization based on features extraction and features tracking, the gyroscope-based stabilization needs significantly less CPU/GPU power. Increasing the image resolution only linearly increases the complexity of our method (due to the image warping);
- Image and video quality do not affect the stabilization process. Stabilization of scenes without features (sea, sand, forest, sky) or image with blur, or any type of image distortions is difficult or impossible for feature-based stabilizations;
- Our stabilization is also independent of weather conditions. When it rains, snows or it is foggy, feature-based stabilization typically struggles with the feature extraction and tracking;
- Gyroscope-based image stabilization is independent of the used camera sensor. The algorithm is the same for visible, SWIR, MWIR or LWIR cameras;
- Changing the zoom does not affect our method, while feature-based methods must be adapted to new features;
- Our method successfully stabilizes images with many moving objects, unlike feature-based stabilization;
- In our method it is easier to separate wanted and unwanted motions (it doesn’t depend on, e.g., moving objects on the scene, parallax, etc.).

Drawbacks:

- Transformation from 3D space to 2D image plane is sensitive to unreliability of the transformation parameters (signals delay, camera intrinsic parameters, gyroscope drift, etc.);
- The stabilization performances depend on the gyroscope technology.

## 6. Conclusions

In this paper, the development of a real-time gyroscope-based video stabilization for electro-optical long-range surveillance systems has been presented. A proposed video stabilization algorithm has been explained in detail, including the transformation from 3D gyroscope movements to 2D image plane, calibration of the transformation parameters. It was argued that the selection of gyroscope is one of the key steps in a design of such systems. When using MEMS gyroscope to increase performance of the stabilization, it was necessary to develop solutions for the problems originating from the measurement noise and bias instability, which are especially pronounced when FOV is narrow. The proposed stabilization method was implemented on a professional surveillance system, and the obtained experimental results were compared with two feature-based digital stabilization techniques. Based on the comparison, we arrived at a conclusion about the advantages and drawbacks of our solution. The advantages include: realization price, computational complexity, and independence of the camera type (sensor spectrum), weather conditions, image distortions or scene content. 

A future work includes development of stabilization methods based on a fusion of the proposed method and digital feature-based methods, which will keep the advantages of both approaches.
